# Natterin-Induced Neutrophilia Is Dependent on cGAS/STING Activation via Type I IFN Signaling Pathway

**DOI:** 10.3390/ijms23073600

**Published:** 2022-03-25

**Authors:** Carla Lima, Aline Ingrid Andrade-Barros, Jefferson Thiago Gonçalves Bernardo, Eniko Balogh, Valerie F. Quesniaux, Bernhard Ryffel, Monica Lopes-Ferreira

**Affiliations:** 1 Immunoregulation Unit of the Laboratory of Applied Toxinology (CETICs/FAPESP), Butantan Institute, Vital Brazil Avenue, São Paulo 05503-009, Brazil; aline.barros@butantan.gov.br (A.I.A.-B.); jefferson.bernardo@butantan.gov.br (J.T.G.B.); monica.lopesferreira@butantan.gov.br (M.L.-F.); 2 MTA-DE Lendület Vascular Pathophysiology Research Group, Research Centre for Molecular Medicine, Faculty of Medicine, University of Debrecen, 4027 Debrecen, Hungary; balogh.eniko@med.unideb.hu; 3 Molecular and Experimental Immunology and Neurogenetics (INEM), UMR7355, CNRS and University of Orléans, 45071 Orléans, France; valerie.quesniaux@cnrs-orleans.fr (V.F.Q.); bernhard.ryffel@cnrs-orleans.fr (B.R.)

**Keywords:** Natterin, neutrophilia, self-DNA, cGAS/STING/IRF3 pathway, type I IFN signaling

## Abstract

Natterin is a potent pro-inflammatory fish molecule, inducing local and systemic IL-1β/IL-1R1-dependent neutrophilia mediated by non-canonical NLRP6 and NLRC4 inflammasome activation in mice, independent of NLRP3. In this work, we investigated whether Natterin activates mitochondrial damage, resulting in self-DNA leaks into the cytosol, and whether the DNA sensor cGAS and STING pathway participate in triggering the innate immune response. Employing a peritonitis mouse model, we found that the deficiency of the *tlr2/tlr4*, *myd88* and *trif* results in decreased neutrophil influx to peritoneal cavities of mice, indicative that in addition to MyD88, TRIF contributes to neutrophilia triggered by TLR4 engagement by Natterin. Next, we demonstrated that *gpcr91* deficiency in mice abolished the neutrophil recruitment after Natterin injection, but mice pre-treated with 2-deoxy-d-glucose that blocks glycolysis presented similar infiltration than *WT* Natterin-injected mice. In addition, we observed that, compared with the *WT* Natterin-injected mice, DPI and cyclosporin A treated mice had a lower number of neutrophils in the peritoneal exudate. The levels of dsDNA in the supernatant of the peritoneal exudate and processed IL-33 in the supernatant of the peritoneal exudate or cytoplasmic supernatant of the peritoneal cell lysate of *WT* Natterin-injected mice were several folds higher than those of the control mice. The recruitment of neutrophils to peritoneal cavity 2 h post-Natterin injection was intensely impaired in *ifnar* *KO* mice and partially in *il-28r* *KO* mice, but not in *ifnγr* *KO* mice. Finally, using *cgas* *KO*, *sting* *KO*, or *irf3* *KO* mice we found that recruitment of neutrophils to peritoneal cavities was virtually abolished in response to Natterin. These findings reveal cytosolic DNA sensors as critical regulators for Natterin-induced neutrophilia.

## 1. Introduction

Natterin proteins were first revealed in the venom of the medically significant Brazilian toadfish, *Thalassophryne nattereri* (V*Tn*), in five orthologs named Natterin (1–4 and -P) [1]. They were identified as toxins since they are responsible for the main damage effects of the V*Tn* envenomation, such as local edema and excruciating pain. Natterin modulates stress levels in the microvasculature, with venous stasis and ischemia that evolves into necrosis [2,3].

We recently performed an extensive screening using available genome databases across a wide range of species and identified 331 species displaying 859 *natterin* or *natterin-like* genes [4]. Structurally, all Natterin-like proteins share a similar architecture with a variable membrane-binding domain in the N-terminal region and a conserved aerolysin-like module [5] in the C-terminal region; the latter contains the AGIP (Ala-Gly-Ile-Pro) family’s signature domain [4].

These proteins containing the Natterin domain are distributed throughout all kingdoms of life, including plants, fungi and sessile marine animals with primitive anatomical structure and organization [4]. However, no homologs have been described in prokaryotes, protists, amphibians and mammals so far. Interestingly, although fish represent the majority of species that contain Natterin-like proteins (109 species with 598 sequences), only four species are venomous and present a venom apparatus, namely, *Plotosus canius*, *Plotosus lineatus*, *Thalassophryne amazonica* and *Thalassophryne nattereri* [5,6].

The presence of a large number of Natterin-like sequences in widely divergent non-venomous species that originated at least 400 million years ago points to the importance of the evolutionary conservation of the aerolysin module [5] across the Natterin group and also shows an important adaptive value consistent with the continuity of the plurality of functions, including action on innate immune defense system, rather than its role only as a toxin.

Natterin founding members are potent pro-inflammatory molecules and a large number of cells may sense and respond to them. Investigations through in vivo studies have showed that Natterin induces local and systemic neutrophilic inflammation in mice dependent on the signals derived from IL-33/ST2 and IL-1β/IL-1R1, as well as IL-1α. Interestingly, the Natterin-dependent neutrophilic inflammation was mediated by the activation of both caspase-1 and caspase-11 by the non-canonical NLRP6 (NOD-like receptor family pyrin domain containing 6) and NLRC4 (NLR family CARD domain containing 4) adaptors through ASC (apoptosis-associated speck like protein) interaction of the inflammasome complex with gasdermin D activation, independent of NLRP3- NOD-, LRR- and the pyrin domain-containing protein 3 [7].

Our data add to previously published studies showing NLRP3 as the only member of the inflammasome family implicated in the sensing of several aerolysin-like pore-forming toxins from several species and emphasize that the NLRP6/NLRC4-dependent neutrophil-mediated response may be part of an innate immune mechanism underlying aerolysin from fish.

Increasing evidence suggests that the stimulator of interferon genes protein (STING) is a critical signaling molecule in immunity and tissue inflammation. Cyclic GMP-AMP (cGAMP) synthase (cGAS) serves as a cytosolic sensor of DNA, and it activates STING to trigger a signaling cascade leading to the production of type I interferons (IFNs) [8]. In addition to pathogen-derived DNA and self-DNA from the nucleus, DNA leaked into the cytosol from damaged mitochondria (mtDNA) activates the cGAS-STING pathway [9]. Furthermore, Swanson et al. [10] demonstrated for the first time that the second messenger, cGAMP, not only activates type I IFNs but also activates the inflammasomes pathway, highlighting the positive cross-talk between inflammasome and cGAS/STING in innate immunity.

Our data demonstrated that Natterin induced neutrophilic inflammation mediated by the activation of the inflammasome complex and that the associated ischemic/necrotic injury could generate the release of the danger-associated molecular pattern (DAMPs). However, the role of the cytosolic DNA–sensing pathway in neutrophilic inflammation induced by Natterin is still unclear. In this work, we investigated whether Natterin-induced inflammation activates mitochondrial damage, resulting in self-DNA leaks into the cytosol and whether the DNA sensor cGAS and STING pathway participate triggering the innate immune response.

## 2. Results

### 2.1. Natterin Induces Signals through TLR4 and MyD88/TRIF Adaptors

**Figure 1:**
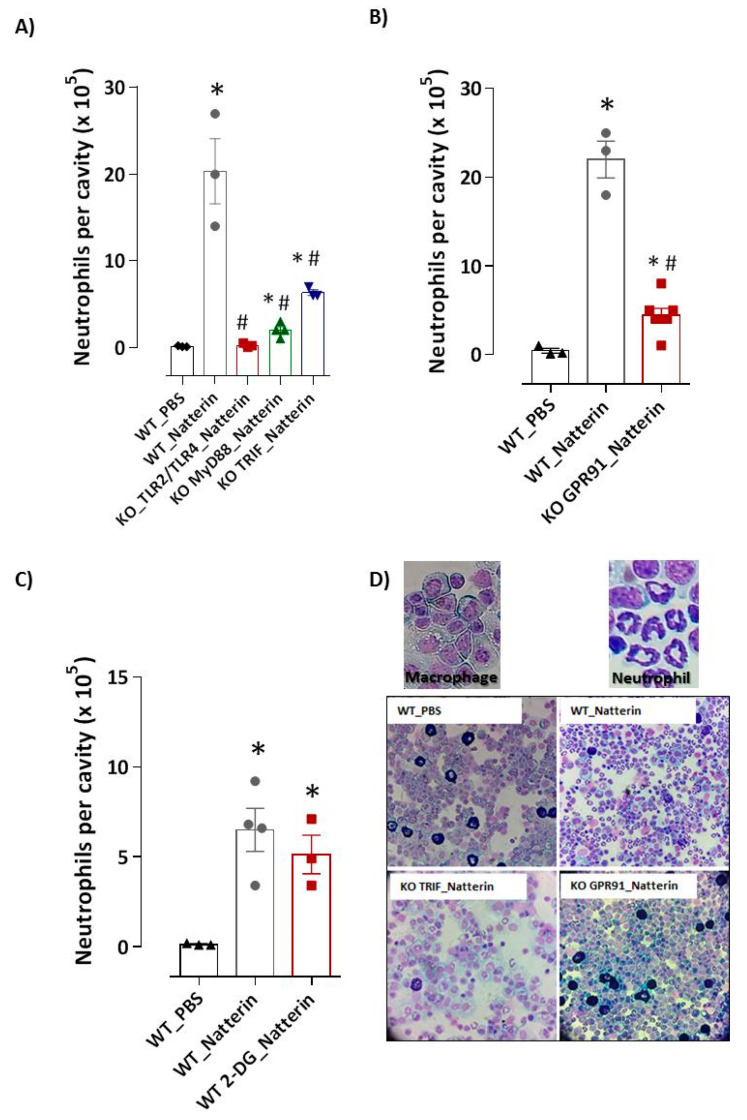
Natterin induces neutrophilic inflammation dependent on PRRs and GPR91 sensors. Natterin (1 μg diluted in PBS) was injected intraperitoneally (i.p.) in non-treated *WT* mice (*WT*_Natterin) or deficient in *tlr2/tlr4*, *myd88* or *trif* (**A**) or *gpr91 KO* mice (**B**) (*KO*_Natterin groups). As a negative control, mice were only injected i.p. with PBS (*WT*_PBS). An independent group of *WT* mice were pre-treated 1 h before Natterin injection with i.p. injection of 2-DG at 10 mg/Kg (*WT* 2-DG_Natterin, (**C**). Two hours after injection, mice were killed and the peritoneal cavities were washed to obtain exudates. Peritoneal exudate cells were harvested and the number of macrophages (large leukocytes with a blue-grey ground glass cytoplasm and an irregularly shaped nucleus with vacuoles.) and neutrophils (with 3–5 nuclear lobes and fine granules within the cytoplasm) was evaluated in cytospin slides stained with a Diff-Quick staining kit. Examples of representative photomicrographs are shown in (**D**). Each bar represents the mean ± SEM of 3–5 animals/group. * *p* < 0.05 compared with *WT*_PBS group and # *p* < 0.05 compared with *WT*_Natterin-group.

The production of inflammatory cytokines that governs the trafficking of leukocytes to organs through the vascular barrier of endothelial cells (ECs) is a result of activation of NFκB, the major outcome of TLR signaling. First, we examined whether neutrophil infiltration was mediated by the engagement of Natterin to pattern-recognition receptors (PRRs). In Figure 1A, we observed that BL6 mice with *tlr2* and *tlr4* gene deficiency presented a drastic reduction (99 ± 0.1%) of neutrophil recruitment to the peritoneal cavity 2 h post-injection, indicating that Natterin engages either TLR2 or TLR4, which induces chemoattractant production for neutrophil recruitment.

TRIF (TIR-domain-containing adapter-inducing interferon-β, encoded by Ticam1) is an adaptor for TLR3 and TLR4; and MyD88 is an adaptor for all TLRs except TLR3, and also is involved in TLR-independent signals activated by IL-1R. Then, we tested the involvement of both adaptors for TLR signaling after Natterin stimulation. In addition, we observed that the recruitment of neutrophil to the peritoneal cavity was significantly decreased by 90% in *myd88 KO* and by 71% in *trif KO* mice 2 h post Natterin injection (Figure 1A,D).

### 2.2. GPR91 Succinate Sensor Drives Neutrophilic Inflammation

Intracellular molecules present in the cytoplasm in the context of major cellular stress could also be detected by intracellular sensors of the innate immune system, either directly or indirectly, and trigger a pro-inflammatory immune response through the formation of inflammasome. Metabolite succinate is a universal metabolic signature response to ischemic/hypoxia conditions [11]. *SUCNR1*/GPR91 is a G protein–coupled (GPCRs) cell surface sensor for extracellular succinate released and accumulated under hypoxia and oxidative stresses [12], and synergizes with TLR, inducing reactive oxygen species (ROS) release [13].

Since the response caused by Natterin is characterized by ischemic and necrotic injury, we hypothesized that the mitochondrial dysfunction with leakage of the intracellular messenger succinate are involved in the neutrophilic mobilization to peritoneal cavity of Natterin-injected mice. Then, with the use of *gpcr91*-deficient mice, we examined whether endogenous succinate accumulation acted as an inflammatory factor triggering neutrophilic infiltration. We found that *gpr91* deficiency led to a strongly reduction (82 ± 0.8%) of neutrophils’ recruitment to peritoneal cavities after Natterin injection in *KO* mice compared to Natterin-injected *WT* mice (Figure 1B,D).

Metabolomics studies have demonstrated that succinate transported from the mitochondria to the cytosol leads to hypoxia inducible factor (HIF)-1α stabilization and a metabolic activity shift [14]. When *WT* mice were pre-treated with 2-deoxy-d-glucose that blocks glycolysis and stimulated with Natterin, no change in the high number of neutrophils was observed compared with Natterin-injected mice (Figure 1*C*). These results suggest that the ischemic accumulation of succinate signaling via the GPR91 receptor plays a decisive role in neutrophilic inflammation, unrelated to the alteration of the metabolic profile for glycolysis.

### 2.3. Mitochondrial Dysfunction Is Important for Natterin-Dependent Neutrophilic Recruitment

**Figure 2:**
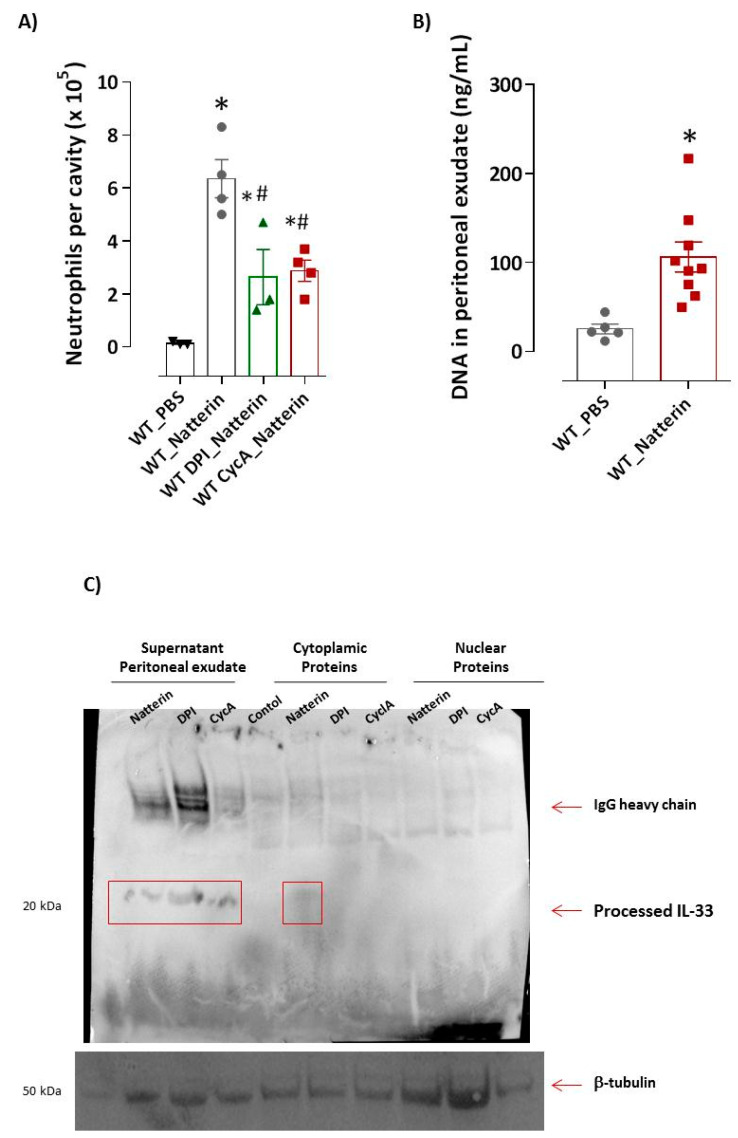
Mitochondrial dysfunction and IL-33 induced in Natterin response. Natterin (1 μg) diluted in PBS was injected i.p. in BL6 *WT* mice previously treated for 1 h with i.p. injection of cyclosporin A at 10 μM (*WT* CycA_Natterin) or DPI at 100 μM (*WT* DPI_Natterin) (**A**). As a negative control, mice were injected i.p. with PBS (*WT*_PBS). As a positive control, mice were only injected with Natterin (*WT*_Natterin). Two hours after injection, mice were killed and the peritoneal cavities were washed to obtain exudates. Peritoneal exudate cells were harvested and the number of neutrophils related to total cell number was evaluated in cytospin slides stained with the Diff-Quick staining kit. Each bar represents the mean ± SEM of 3–5 animals/group. * *p* < 0.05 compared with *WT*_PBS group and # *p* < 0.05 compared with *WT*_Natterin group. Concentrated supernatant of peritoneal exudates from *WT*_PBS or *WT*_Natterin groups of mice were analyzed for the content of double-stranded DNA using Quant-iTPicoGreen dsDNA reagent (**B**). Proteins present in concentrated supernatant or cytoplasmic and nuclear proteins (**C**) collected 2 h after Natterin injection were analyzed using the iBind^TM^ Flex Western System with goat anti-mouse IL-33 (processed form: 18 to 20 kDa) followed by the secondary antibody anti-goat IgG-HRP. The β-tubulin was used as housekeeping protein. The immune complex was revealed by enhanced chemiluminescence detection system.

Accumulated succinate is rapidly re-oxidized by succinate dehydrogenase, driving extensive mitochondrial ROS generation, a critical early driver of injury [15]. Next, we wanted to identify whether ROS production induced by Natterin is involved in the neutrophilic infiltration. Figure 2A shows a decreased (59 ± 2%) infiltration in neutrophils to peritoneal cavities of mice pre-treated with DPI (diphenyleneiodonium), a potent inhibitor of nitric oxidase synthase (NADPH), which blocks mitochondrial ROS and phagosomal ROS [16].

Ischemic injury is mediated, at least in part, by the opening of the mitochondrial permeability transition pore (mPTP) with subsequent mitochondrial swelling, rupture and release into the cytosol of other DAMPs besides succinate, such as circular mtDNA, that can be degraded and released into the cytoplasm [11]. DAMPs exert an important role for neutrophil inflammation [17,18].

In Figure 2A, it can be observed that mice pre-treated with cyclosporin A [19], an inhibitor of mPTP opening via binding to mitochondrial peptidyl-prolyl cis-trans isomerase F (PPIF, also known as cyclophilin D) presented 55% of reduction in the number of neutrophils in peritoneal exudate compared with *WT* Natterin injected-mice.

Self-derived dsDNA, including linear nuclear DNA and mtDNA into the extracellular space, where it can be engulfed and sensed by endosomal or cytoplasmic nucleic acid sensors, elicits neutrophilic inflammation [20]. Interestingly, we found that the levels of dsDNA in the supernatant of the peritoneal exudate of *WT* Natterin-injected mice were several folds higher (4-fold) than those of the control mice (Figure 2B).

IL-33 is a nuclear-targeted cytokine abundantly expressed at mucosal barriers, which can be released from intact cells to propagate inflammation [21,22]. Interestingly, the role for cleaved IL-33 alarmin decorating NETs in human systemic lupus erythematosus, linking neutrophil activation, type I IFN production and end-organ inflammation has been demonstrated [23].

Next, we identified the subcellular location of IL-33 after Natterin stimulation in processed samples of neutrophil-rich peritoneal cavity exudates. Increased processed IL-33 (20 kDa) was observed in the exudate supernatant or cytoplasmic supernatant of peritoneal exudate (obtained after lysis of the cell pellet from neutrophil-rich Natterin-injected mice), but not in the nuclear samples (Figure 2C). Moreover, mice pre-treated with DPI or cyclosporin A continued to release processed IL-33 in the supernatant of peritoneal exudate after Natterin stimulation, showing that the release of the processed cytokine by activated neutrophils is an independent event of mPTP opening or ROS production.

### 2.4. cGAS/STING/IRF3 via Type I IFN Axis Supports Natterin-Neutrophilic Inflammation

Our previous results demonstrated that caspase-1 and caspase-11 were required for the processing of pro-IL-1β, which together with IL-1α, control the local and systemic neutrophilic inflammation in response to Natterin [7]. Type I IFNs induce caspase-11 expression, an event that is both necessary and sufficient to promote caspase-11 auto-processing. Yi [24] summarized and discussed the current studies exploring the activation mechanisms and the regulatory roles of non-canonical inflammasomes, such as mouse caspase-11 and human caspase-4 and caspase-5 non-canonical inflammasomes in the inflammatory response and human diseases.

**Figure 3:**
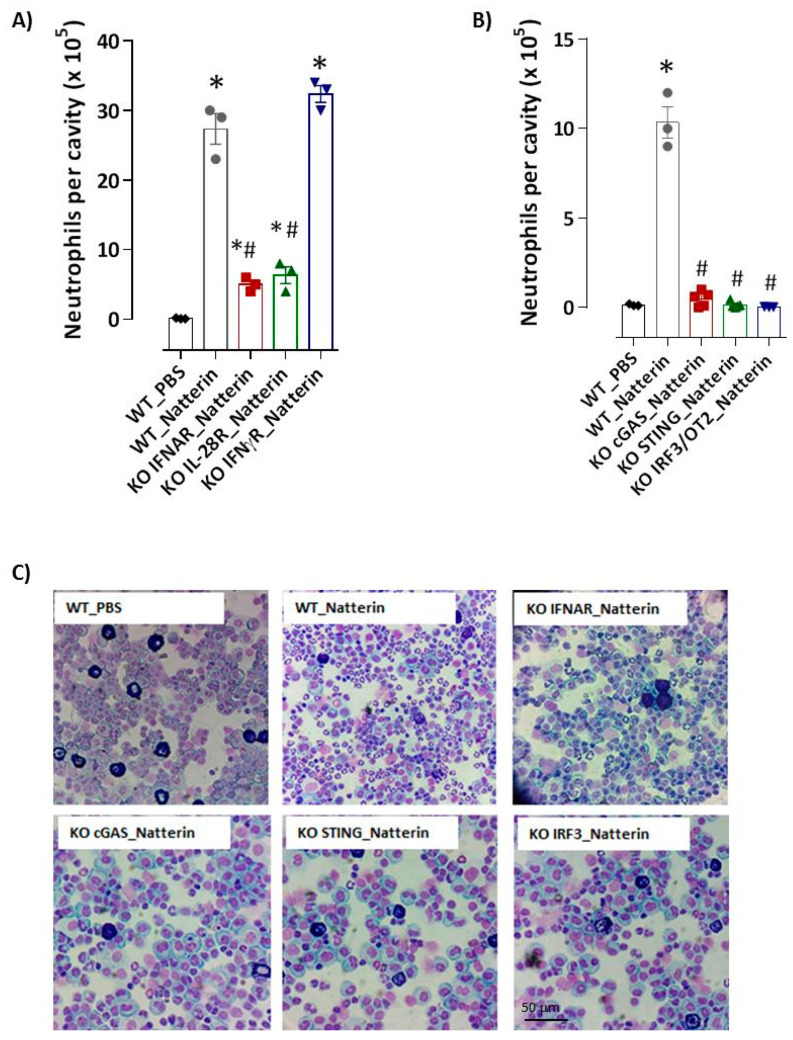
cGAS/STING via type I IFN axis supports the neutrophilic inflammation. Natterin (1 μg) diluted in PBS was i.p. injected into BL6 *WT* (*WT*_Natterin) or in *ifnar*-, *il-28r*-, or *ifnγr*-deficient mice (**A**), or in *cgas*-, *sting*- or *irf3*-deficient mice (*KO*_Natterin groups) (**B**). Two hours after Natterin injection, mice were killed and the peritoneal cavities were washed to obtain exudates. Peritoneal exudate cells were harvested and the number of neutrophils related to total cell number was evaluated in cytospin slides stained with the Diff-Quick staining kit. Examples of representative photomicrographs are shown in (**C**). Each bar represents the mean ± SEM of 3–5 animals/group. * *p* < 0.05 compared with *WT*_PBS group and # *p* < 0.05 compared with *WT*_Natterin-group.

Here, we interrogated the upstream regulation of capase-11, focusing on type I (IFN α/β) or III (λ) IFNs signaling. Notably, recruitment of neutrophils to peritoneal cavity 2 h post-Natterin injection was intensely impaired in *ifnar KO* mice, which are deficient in the type I interferon-α/β receptor (75 ± 1%) or even partially in *il-28r KO* mice, deficient in the IFNλ receptor (69 ± 2%) (Figure 3A,C). In addition, using *ifnγr KO* mice, we confirmed that the neutrophilic infiltration following Natterin stimulation is negatively regulated by IFNγR signaling (Figure 3A).

One way to activate the type I IFN signaling response [25,26] and IL-33 release [27,28] is via the cGAS/STING pathway. Therefore, we assessed the Natterin-induced STING pathway’s downstream molecules’ requirement, such as the transcription factor interferon regulatory factor 3 (IRF3), in neutrophilic inflammation.

Using *cgas KO*, *sting KO* or *irf3 KO* mice we found that recruitment of neutrophils to peritoneal cavities was virtually abolished (95, 99 and 99 ± 0.1%) in those mice in response to Natterin (Figure 3B,C).

## 3. Discussion

Recently, we investigated the regulatory mechanisms controlling acute neutrophilic inflammation induced by Natterin, a family of proteins responsible for the toxic effects of the venom of *Thalassophryne nattereri*. We reported that Natterin induced the extracellular release of mature IL-1β and the sustained production of IL-33 by bronchial epithelial cells, which are essential signals for driving local and systemic neutrophil migration [7]. In addition, our data showed that the IL-1β-dependent neutrophilic inflammation induced by Natterin is the result of non-canonical activation of the inflammasome complex with the participation of cytosolic NLRP6/NLRC4 sensors.

In this study, we have provided evidence that STING is an important signaling molecule in IL-1β-dependent neutrophilic inflammation mediated by inflammasome activation in response to Natterin. In fact, our results indicated that Natterin leads to the release of significant amount of DNA in peritoneal exudate, activates cGAS, STING and IRF3, which mediates neutrophilic inflammation. In *cgas*-, *sting*- and *irf3*-deficient mice, the influx of neutrophils was alleviated.

The inflammasome and type I IFNs pathways are two seminal routes by which innate immunity is activated to combat a wide variety of microbial pathogens. Mitochondrial DNA fragments serve as one of the ligands that cause STING activation, resulting in the activation of IRF3 and NF-κB and the expression of type I IFNs and other pro-inflammatory genes [29,30,31,32].

Although the Natterin-dependent mechanisms of cellular activation are still poorly understood, in recent years, considerable advances have been made regarding the identification and characterization of aerolysin-mediated damage. Such studies have highlighted the underlying sequential nature, including the recognition as antigen by PRRs in immune cells [33,34] resulting in activation and production of pro-inflammatory molecules [35], and the binding specifically to GPI-anchored proteins at the surface of target cells promoting pore formation and cytosol insertion of the toxin [36]. Accordingly, pore formation triggering further potassium efflux and calcium influx may enable the secretion of cytokines and occurs downstream of p38 mitogen-activated protein kinases (MAPK), inflammasome activation, caspase-1 processing and activation of IL-1β secretion [36].

Here, we revealed that the deficiency of the *tlr2*/*tlr4*, *myd88* and *trif* results in decreased neutrophil influx to peritoneal cavities of mice, indicative that in addition to MyD88, TRIF contributes to neutrophilia triggered by TLR4 engagement by Natterin. We can attribute the importance of TRIF in Natterin-induced neutrophilia to its role as an inducer of IL-33 production and as an alternate TRIF-IRF3-axis–mediated IFN-β induction [27]. TLR9 is activated in response to DNA. However, the impact of TLR9 signaling on Natterin-induced neutrophilic inflammation remains to be determined.

Interestingly, the role for cleaved IL-33 alarmin decorating NETs in human systemic lupus erythematosus, linking neutrophil activation, type I IFN production and end-organ inflammation has been recently demonstrated by Georgakis et al. [23]. Ozasa et al. [28] found that IRF3/7, which are signal transducers downstream of TBK1, are required for IL-33 release from lung fibroblasts in response to cGAMP, which functions as an allergy-prone adjuvant inducing strong type-2 immune responses to co-inhaled allergen in the airway.

These findings fit with our model that identified the production of IL-33 by bronchial epithelial cells and the dependence of ST2/IL-33 signaling on the local and systemic neutrophil migration in response to Natterin [7].

Studies have shown that activation of the STING pathway requires TRIF that interacts directly with STING to promote its dimerization and membrane translocation [25,26]. Previously, Yamamoto, Sato and Hemmi [37] demonstrated a prominent feature of TRIF-dependent IRF signaling in the production of type I IFN. More recently, a hemorrhagic shock model confirmed that the deficiency of the TLR4 and its intracellular adaptor TRIF results in decreased activation of STING’s downstream mediators, TBK1 and IRF3, and the expression of type I IFNs [38].

A recent study with peripheral blood from asthmatic patients revealed that increased STING expression may be associated with exacerbation of the disease [39]. Furthermore, Han et al. [40] described the accumulation of cytosolic dsDNA and cGAS-dependent cytokine production in IL-33-stimulated human bronchial cells and in mice submitted to three different allergic airway inflammation protocols, highlighting the important role of IL-33 induced cytosolic dsDNA accumulation and cGAS/STING pathway activation to asthma pathogenesis.

In our current study, we found the importance of type I (IFN α/β) or III (λ) IFNs signaling in non-canonical inflammasome-dependent neutrophilic inflammation, since mice deficient in IFNAR or IL-28R receptors had a significant reduction in neutrophil recruitment in response to Natterin. Indeed, we found an opposite effect of type II FN (γ) over type I and III IFNs in the regulation of the number of infiltrating neutrophils, corroborating the findings that report the negative reciprocal counter-regulation. Saikh [41] describe that MyD88 up-regulation with many viral infections is linked to decreased antiviral type I IFN response, and MyD88 exert an inhibitory effect on the TRIF-mediated downstream signaling pathway of the type I IFN response.

cGAS serves as a cytosolic sensor of dsDNA and it activates STING, leading to a type I IFN response via synthesis of the secondary messenger, cGAMP [42]. Together, our data that show the requirement of cGAS for Natterin-induced neutrophilic inflammation confirm the crucial importance of cGAS-STING-IRF3 axis as a common pathway.

There are several possible mechanisms by which mtDNA leaks into the cytosol to induce cGAS-STING signaling-mediated inflammation. First, it has been described that IL-1β signaling causes DNA damage and self-DNA release [9,43]. ROS can induce oxidative mitochondrial damage, resulting in mtDNA leaks into the cytosol [44]. Additional contributions for the leakage of self-DNA come from increased or prolonged mPTP opening in activated neutrophils or from dead or stressed cells [20].

**Figure 4:**
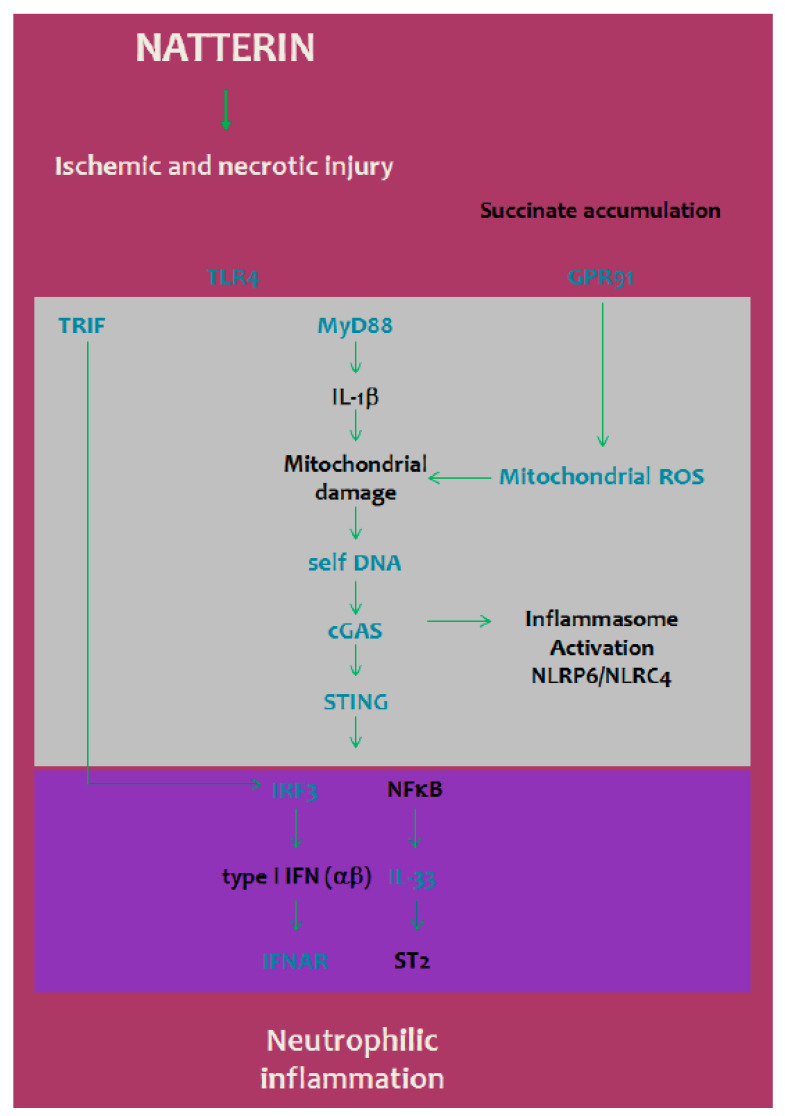
Neutrophilic inflammation induced by Natterin requires cGAS/STING/IRF3 via type I IFN receptor. Natterin induces neutrophilic infiltration with cell activation and release of cytosolic molecules, such as DNA, succinate and ROS. cGAS/STING drives IRF3-mediated inflammation dependent on type I IFN receptor. The activation of the STING pathway requires TLR4/TRIF-dependent pathway, essential for the production of type I IFNs, which synergizes with processed IL-33 to coordinate inflammation. Our data clarify that the neutrophilic inflammation induced by Natterin an aerolysin-like toxin is the result of activation of cytosolic DNA sensors pointing to the possibility of new pharmacological tools for its control.

We observed that, compared with the *WT* Natterin-injected mice, *gpcr91 KO* mice or DPI and cyclosporin A treated-mice had a lower number of neutrophils in the peritoneal cavity, implying that the ischemic accumulation of succinate-dependent ROS production plays a decisive role in neutrophilic inflammation. Interestingly, the released DNA in our model seems to be associated to a mechanism partially dependent of mPTP opening. Together, these data suggest that Natterin is a potent trigger for mitochondrial damage and mtDNA leakage into the cytosol, which activates the cGAS cytosolic DNA sensor that causes STING signaling, driving IRF3-mediated secretion of type I IFNs, which synergizes with IL-33 to promote neutrophilic inflammation (Figure 4). Our study indicates a sophisticated interplay between cGAS/STING and type I IFN pathways that connect the non-canonical pathways of inflammasome activation for the regulation of neutrophilia in response to Natterin.

## 4. Materials and Methods

### 4.1. Mice

Male 7–8-week-old C57BL/6J wild-type (BL6 *WT*) or TLR2/4-, TRIF-, MyD88-, IFNAR-, IFNγR-, IRF-3/OT2-, cGAS-, STING-, GPR91- and IL-28R-deficient mice—*KO* (all on a BL6 background) were maintained at UPS44 TAAM (Orleans, France) under SPF conditions, and were housed in positive-pressure air-conditioned units (25 °C, 50% relative humidity) on a 12 h light/dark cycle. This study was carried out in strict accordance with the recommendations in the Guide for the Care and Use of Laboratory Animals of the Brazilian and French Ethics Committee. The protocol was approved by the Committee on the Ethics of Animal Experiments of the Butantan Institute (no. 9381060819) and of CNRS Campus Orleans (CCO) under numbers CLE CCO 2015-1087 and approved by the French Minister under APAFIS #19361.

### 4.2. Natterin Preparation

*T. nattereri* fish venom was obtained from fresh captured specimens at the Mundau Lake in the state of Alagoas, Brazil, with a trawl net from the muddy bottom of lake. Fish were transported to the Immunoregulation Unit of Butantan Institute according to the Brazilian Environmental Agency (IBAMA—Instituto Brasileiro do Meio Ambiente e dos Recursos Naturais Renováveis) under the license no. 16221-1. Venom was immediately extracted from the openings at the tip of the spines by applying pressure at their bases. After centrifugation, venom was pooled and stored at −80 °C before use. After that, fish were anesthetized with 2-phenoxyethanol prior to sacrifice by decapitation. The purified 35–38-kDa Natterin solution from *T. nattereri* fish venom was prepared with a pool of venom collected in different months of the year in Alagoas according to Komegae et al. [33]. The venom was fractionated by cation exchange chromatography, using the fast protein liquid chromatography system (FPLC—Pharmacia, Uppsala, Sweden). Immediately before chromatography, 2 mg venom was diluted in 500 μL of buffer A (20 mM Tris-hydroxymethylaminomethane, pH 8.3) and the solution centrifuged at 10,000× *g* for 5 min. The sample was applied on Mono S column HR 5/5 equilibrated with buffer A. The retained proteins were eluted with a linear gradient of NaCl (sodium chloride) 0–2 M and collected at a flow rate of 1 mL/min. The elution profile was determined by measuring absorbance at 280 nm. Fractions 1–4, except the 5th, corresponding to the Natterins, were pooled (referred to as Natterin), dialyzed against 50 mM Tris/HCl pH 7.4 and evaluated with respect to its protein content and kept at −20 °C until use. The obtained Natterin were analyzed by polyacrylamide gel electrophoresis with 12% SDS (SDS-PAGE). Endotoxin content resulting in a total dose <0.8 pg LPS was evaluated with chromogenic Limulus amoebocyte lysate assay (no. QCL-1000, Bio-Whittaker) according to the manufacturer’s instructions.

### 4.3. Acute Inflammation Induced by Natterin and Pharmacological Treatments

Non-treated mice (*n* = 3 to 5/group) were intraperitoneally (i.p.) injected with Natterin at 1 μg in 500 μL (*WT*_Natterin or *KO*_Natterin groups). Non-treated *WT* mice i.p. injected only with 500 μL of PBS were considered as the negative control group (*WT*_PBS). Independent groups of *WT* mice were pre-treated 1 h before Natterin stimulation with i.p. injection of 500 μL of diphenyleneiodonium at 100 μM (*WT* DPI_Natterin group, no. D2926, Sigma-Aldrich Chemie GmbH, Taufkirchen. Germany); cyclosporin A at 10 μM (*WT* CycA_Natterin group, no. 12088, Cayman Chemical, MI, USA); and 2-deoxy-d-glucose at 10 mg/kg (*WT* 2-DG_Natterin group, no. D8375, Sigma-Aldrich Chemie GmbH).

### 4.4. Peritoneal Cell Suspension Collection

After 2 h, mice were sacrificed by isoflurane inhalation, their peritoneal cavities were washed with 2 × 2.5 mL of cold PBS and the exudates harvested were centrifuged at 1500 rpm at 4 °C for 10 min. According to Santos et al. [45], total leukocyte counts were performed using a hemocytometer and cytocentrifuge slides containing 100 μL of cell suspension were prepared, air dried, fixed in methanol and stained with the Diff-Quick staining set, and analyzed in an optical microscope a 40× objective. For differential cell counts, 300 leukocytes were classified as macrophages or polymorphonuclear neutrophils and counted, based on staining and morphological characteristics, using a light microscope Axio Imager A1 (Carl Zeiss, Jena, Germany) with an AxioCam ICc1 digital camera (Carl Zeiss).

### 4.5. Double-Stranded DNA Content Measurement

The supernatants obtained after peritoneal exudate centrifugation were precipitated for protein concentration by 12 h of incubation at −20 °C with acetone. The concentrated supernatant was analyzed for the content of double-stranded DNA using Quant-iTPicoGreen dsDNA reagent (no. P11495, Invitrogen, Carlsbad, CA, USA), according to Nascimento et al. [46].

### 4.6. Western Blot

The cell pellets collected from the peritoneal exudate were resuspended in lysis buffer solution (RIPA no. 9806, cell signaling added of Pierce protease and phosphatase inhibitor no. 88668, Thermo Fisher Scientific, MA, USA), and kept for 30 min on ice and sonicated for 3 s × 10 amplitude at 4 °C. It was then centrifuged at 14,000 rpm at 4 °C for 15 min. The collected supernatant was precipitated in acetone to obtain the cytoplasmic proteins and the pellets were resuspended in lysis buffer solution and kept for more 30 min on ice followed by 5 min of immersion bath in liquid nitrogen. After centrifugation at 14,000 rpm at 4 °C for 15 min, the collected supernatant was precipitated in acetone to obtain the nuclear proteins.

Samples (exudate supernatant, cytoplasmic and nuclear proteins) were incubated in sample buffer (Novex 4×, no. B0007, Thermo Fisher Scientific, MA, USA) with or without reducing agent (Novex 10×, no. B0009, Thermo Fisher Scientific) for 10 min at 70 °C and applied to 8–16% Precise gel (no. 0025203, Thermo Fisher Scientific) in a Bolt Mini Gel Tank system (no. B4477599, Novex, Thermo Fisher Scientific) and power supply EPS601 (G&E Sigma-Aldrich Chemie GmbH) submitted to running during 45 min under 165 V, 200 mA and 100 W in 1× MES buffer (Novex 20×, no. B0002, Thermo Fisher Scientific).

After running, the proteins were transferred to a nitrocellulose membrane (Novex, no. IB301001, Thermo Fisher Scientific) using the iBlot Dry Blotting system (no. 10072147, Invitrogen Thermo Fisher Scientific, Waltham, MA, USA) for 7 min or by the traditional transfer method using Mighty Small Transphor (Sigma-Aldrich Chemie GmbH, no. 80620426). Specific proteins were detected according to Grund et al. [47] using the goat anti-mouse IL-33 (processed form: 18 to 20 kDa, no. 842875 at 1 μg/mL, R&D Systems, Inc., Minneapolis, MI, USA) followed by the second antibody donkey anti-goat IgG-HRP (no. sc 2033 at 1/2000, Santa Cruz Biotechnology Inc., Dallas, TX, USA) and anti-β-tubulin (no. IMG-5810A at 0.6 μg/mL, IMGENEX, Novus Biologicals LLC, Littleton, CO, USA) followed by secondary antibody mouse anti-rabbit IgG HRP True Blot (no. 18-8816-33 at 1/500, Rockland, Hamburg, Germany) for 3 h on the iBindTM Flex Western System apparatus using iBind Cards (Invitrogen Thermo Fisher Scientific, no. SLF2010). The development was performed by adding an ECL chemiluminescence kit (Novex, no. WP20005, Thermo Fisher Scientific) mixing equal parts of substrates solution. Using the UVITEC, model Alliance 2.7-89-EPI/20K, chemiluminescent bands were detected at 5 min exposure time. Any intensification or color lightening tools were applied to the gels.

### 4.7. Statistical Analysis

All values were expressed as mean ± SEM. Experiments using 3 to 5 mice per group were performed independently during the last two times of this study. Parametric data were evaluated using analysis of variance, followed by the Bonferroni test for multiple comparisons. Non-parametric data were assessed using the Mann–Whitney test. Differences were considered statistically significant at *p* < 0.05 using the Graph Pad Prism software (Graph Pad Software, v6.02, 2013, La Jolla, CA, USA).

## Data Availability

Not applicable.
